# Landscape of the Peripheral Immune Response Induced by Intraoperative Radiotherapy Combined with Surgery in Early Breast Cancer Patients

**DOI:** 10.1002/advs.202308174

**Published:** 2024-11-04

**Authors:** Danian Dai, Xuerui Li, Hongkai Zhuang, Yun Ling, Lezi Chen, Cheng Long, Jinhui Zhang, Yunjie Wang, Yuehua Li, Hailin Tang, Bo Chen

**Affiliations:** ^1^ Department of Plastic and Peripheral Vascular Surgery Guangdong Provincial People's Hospital (Guangdong Academy of Medical Sciences) Southern Medical University Guangzhou Guangdong 510080 China; ^2^ Department of Breast Cancer Cancer Center Guangdong Provincial People's Hospital (Guangdong Academy of Medical Sciences) Southern Medical University Guangzhou Guangdong 510080 China; ^3^ Guangdong Provincial Key Laboratory of Malignant Tumor Epigenetics and Gene Regulation Sun Yat‐sen Memorial Hospital Sun Yat‐Sen University Guangzhou Guangdong 510120 China; ^4^ Department of Breast Surgery The Second Affiliated Hospital of Guangzhou Medical University Guangzhou Guangdong 510260 China; ^5^ Department of Pathology Yueyang Maternal Child Health‐Care Hospital Yueyang Hunan 414000 China; ^6^ State Key Laboratory of Oncology in South China Guangdong Provincial Clinical Research Center for Cancer Sun Yat‐Sen University Cancer Center Guangzhou Guangdong 510060 China; ^7^ School of Medicine Hunan University of Chinese Medicine Changsha Hunan 410208 China; ^8^ Department of Oncology, The First Affiliated Hospital Hengyang Medical School University of South China Hengyang Hunan 421001 China

**Keywords:** breast cancer, intraoperative radiotherapy, peripheral immune response, single‐cell RNA sequencing, single‐cell T cell receptor sequencing

## Abstract

A comprehensive analysis of the immune response triggered by intraoperative radiation therapy (IORT) remains incomplete. In this study, single‐cell RNA sequencing and single‐cell T cell receptor sequencing are conducted on peripheral blood mononuclear cells (PBMCs) from patient with early‐stage breast cancer before and after IORT. Following IORT combined with surgery (defined as IORT+Surgery), PBMC counts remained stable, with increased proportions of T cells, mononuclear phagocytes, and plasma cells, and a reduction in neutrophil proportions. The cytotoxic score of CD8Teff_GZMK cells increased significantly post‐IORT. Communication between CD8Teff_GZMK cells and other immune cells via MIF_CD74 and MIF_TNFRSF14 is decreased after IORT. cDCs showed an upregulation of the MCH II signaling pathway, while memory B cells exhibited enhanced activation of the B cell pathway. T cell clones expanded significantly after treatment. IORT+Surgery demonstrated the ability to partially suppress the anti‐tumor effects of neutrophils. Flow cytometry analysis and co‐culture experiments are utilized to delve deeper into the functional alterations in T cells. IORT+Surgery significantly enhanced T cell cytotoxic activity. Blockade of PD‐1 of post‐IORT PBMCs shows higher T‐cell activity than that of pre‐IORT PBMCs. This research highlights IORT's impact on immune cells, offering insights for targeting immune responses in breast cancer.

## Graphical abstract

This study investigates the immune response to intraoperative radiation therapy (IORT) in early‐stage breast cancer using single‐cell RNA and T cell receptor sequencing. IORT combined with surgery increases T cell cytotoxicity, alters immune cell communication, and modulates T cell clonal expansion, offering insights into immune targeting strategies for breast cancer.

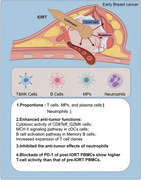

## 1 Introduction

The development of screening technology has improved the detection rate of early breast cancer.^[^ ^1^ ^]^ Breast‐conserving surgery with external radiotherapy has become a standard approach for treating early‐stage breast cancer. Radiotherapy contributes to enhancing both local disease management and overall survival rates.^[^ ^2^ ^]^ Postoperative adjuvant radiotherapy for breast conserving surgery is performed after wound healing or completion of chemotherapy. The routine plan for whole breast radiotherapy is 50Gy/25 times, and the routine plan for tumor bed pushing is 10–16Gy/5‐8 times.^[^ ^3^ ^]^ The total treatment time takes 5–7 weeks. Radiotherapy causes inconvenience and increases economic cost for patients. Some patients might experience delays in radiotherapy due to postoperative complications. Intraoperative radiotherapy (IORT) is a high irradiation dose delivered during a surgical procedure.^[^ ^4^ ^]^ IORT avoids the deviation of tumor bed positioning caused by tissue flap displacement after surgery, and improves the accuracy of tumor bed push radiotherapy. IORT offers benefits in effectively managing the tumor bed, which represents the region with an elevated risk of recurrence. It also reduces the risk of tumor proliferation caused by delayed radiotherapy due to chemotherapy. There are similar results in terms of survival and toxicity for IORT treatment as for whole‐breast radiation therapy, but it takes less time to complete the treatment. In this context, IORT presents a favorable choice for early‐stage breast cancer patients undergoing breast‐conserving surgery.

It's important to note that the tumor microenvironment (TME) is influenced by a multitude of factors. Genetic alterations, epigenetic modifications, and interactions between different cell types contribute to the complexity of TME regulation.^[^ ^5^, ^6^, ^7^ ^]^ Additionally, radiotherapy can alter cell phenotype and tissue composition, thereby impacting both cancer cell survival and the tumor microenvironment.^[^ ^8^ ^]^ Radiation affects not only targeted cells but also non‐irradiated neighboring cells, a phenomenon referred to as radiation‐induced bystander effects. In some published studies, IORT has been shown to affect tumor cells and wound fluid.^[^ ^9^, ^10^ ^]^ A single high dose of IORT induces immunogenic cell death of cancer cell by triggering the immune system.^[^ ^11^ ^]^ Galiana et al. conducted a flow cytometry examination and observed a significant rise in the NK CD56+high CD16+ subpopulation three weeks after IORT.^[^ ^12^ ^]^ However, information regarding the peripheral immune response triggered by IORT remains unknown.

Single‐cell RNA sequencing (scRNA‐seq) can comprehensively analyze the immune system in an unprecedented way.^[^ ^13^, ^14^ ^]^ By using scRNA‐seq, it is possible to characterize cell types, their functions, and the heterogeneity of their compositions.^[^ ^15^, ^16^, ^17^ ^]^ To clarify the processes of the immune reaction triggered by IORT, we employed scRNA‐seq to analyze the immune response in peripheral blood mononuclear cells (PBMCs) collected from early‐stage breast cancer patients both before and after IORT combined with surgery (defined as IORT+Surgery). A high‐resolution transcriptomic landscape of blood immune cell subsets was depicted in our study. In this study, we have identified several significant findings: 1) Following IORT, PBMC counts remained stable, while the proportions of T cells, monocytes (MPs), and plasma cells increased, accompanied by a decrease in neutrophil proportions. 2) Intraoperative radiotherapy significantly enhanced the anti‐tumor functions of peripheral immune cells. Notably, it resulted in a substantial increase in the cytotoxic activity of CD8Teff_GZMK cells. Furthermore, we detected an upregulation of the MCH II signaling pathway in cDCs and enhanced activation of the B cell pathway in memory B cells. 3) T cell clones experienced substantial expansion following intraoperative radiotherapy. 4) Intraoperative radiotherapy demonstrated the ability to partially inhibit the anti‐tumor effects of neutrophils. 5) Blocking PD‐1 in post‐IORT PBMCs resulted in higher T‐cell activity compared to that in pre‐IORT PBMCs.

## 2 Results

### 2.1 Basic Information of Patients and Analysis of Single Immune Cell Profiling

**Figure 1:**
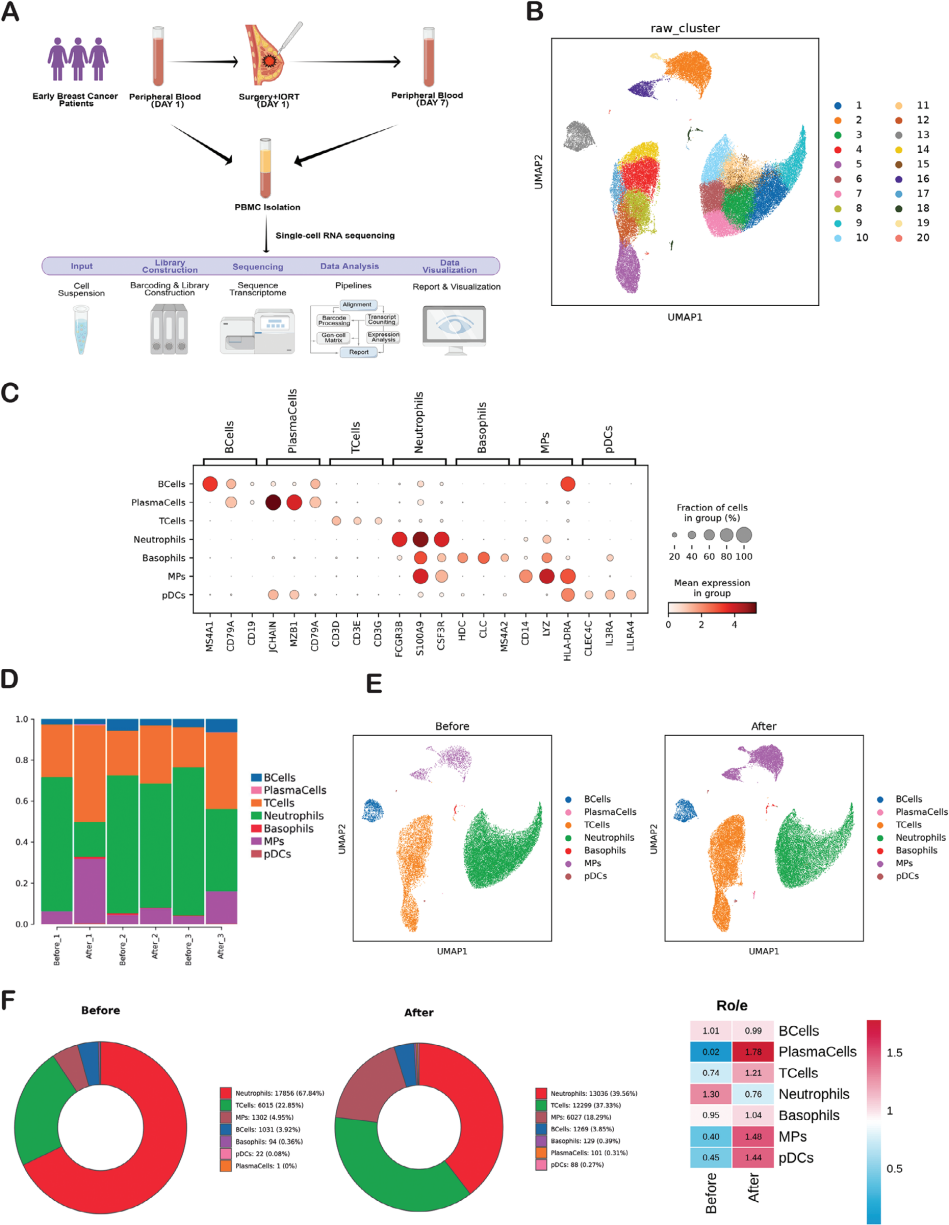
ScRNA‐seq profiling of peripheral immune response induced by IROT of early breast cancer. A) Schematic representation of the scRNA‐seq strategy. B)Visualization of cell clusters across all single cells through a Uniform Manifold Approximation and Projection (UMAP) plot. Twenty cell clusters are displayed based on marker gene expression levels. C) The DotPlot representation showcasing the unbiased gene expression profiles obtained from single‐cell RNA sequencing clusters. The top three genes within each cluster, identified by log‐fold change, are presented. Dot size indicates the percentage of cells within a cluster expressing the respective gene; dot color reflects expression level. D) Distribution of cell cluster proportions across different samples. E) UMAP plots showing changes of peripheral immune cells induced IORT+Surgery of breast cancer. F) Pie charts depicting the proportions of each cell cluster before and after IORT combined with surgery. Ratio of observed cell number to expected cell number revealed by Ro/e.

We collected peripheral blood samples from three early breast cancer patients both before and one week after IORT. All three patients underwent breast‐conserving surgery for breast cancer, with negative margins confirmed on pathological examination. Subsequently, we conducted scRNA‐seq and single‐cell paired TCR analysis (**Figure** **1**A). Among these three patients, two exhibited the ER+/HER2‐ subtype, while one presented with the HER2+ subtype. The clinicopathological characteristics of the three patients and their routine blood test results before breast conserving surgery are presented in Tables S1 and S2 (Supporting Information). The transcriptomic profiles of 59270 cells were obtained following standard data processing and quality control procedures. High‐quality filtering was performed on this dataset. We examined the distribution of peripheral immune cells by considering the expression of canonical lineage markers and other genes that were specifically upregulated within each cluster (Figure 1B,C). 59270 cells were annotated into seven major categories, including 18314 T cells, 2300 B cells, 102 Plasma cells, 30892 Neutrophils, 7329 MPs, 110 pDCs and 223 Basophils (Table S3, Supporting Information). The histogram depicting the general distribution of peripheral immune cells in each sample (Figure 1D) After IORT+Surgery, patients exhibited an elevated proportion of Plasma cells, T cells, and MPs, along with a reduced proportion of Neutrophils, while B cell levels remained stable in peripheral blood (Figure 1E,F). However, it is worth noting that the absolute number of these cells did not exhibit significant changes.

### 2.2 An overview of T cells and NK Cells in the Peripheral Blood

**Figure 2:**
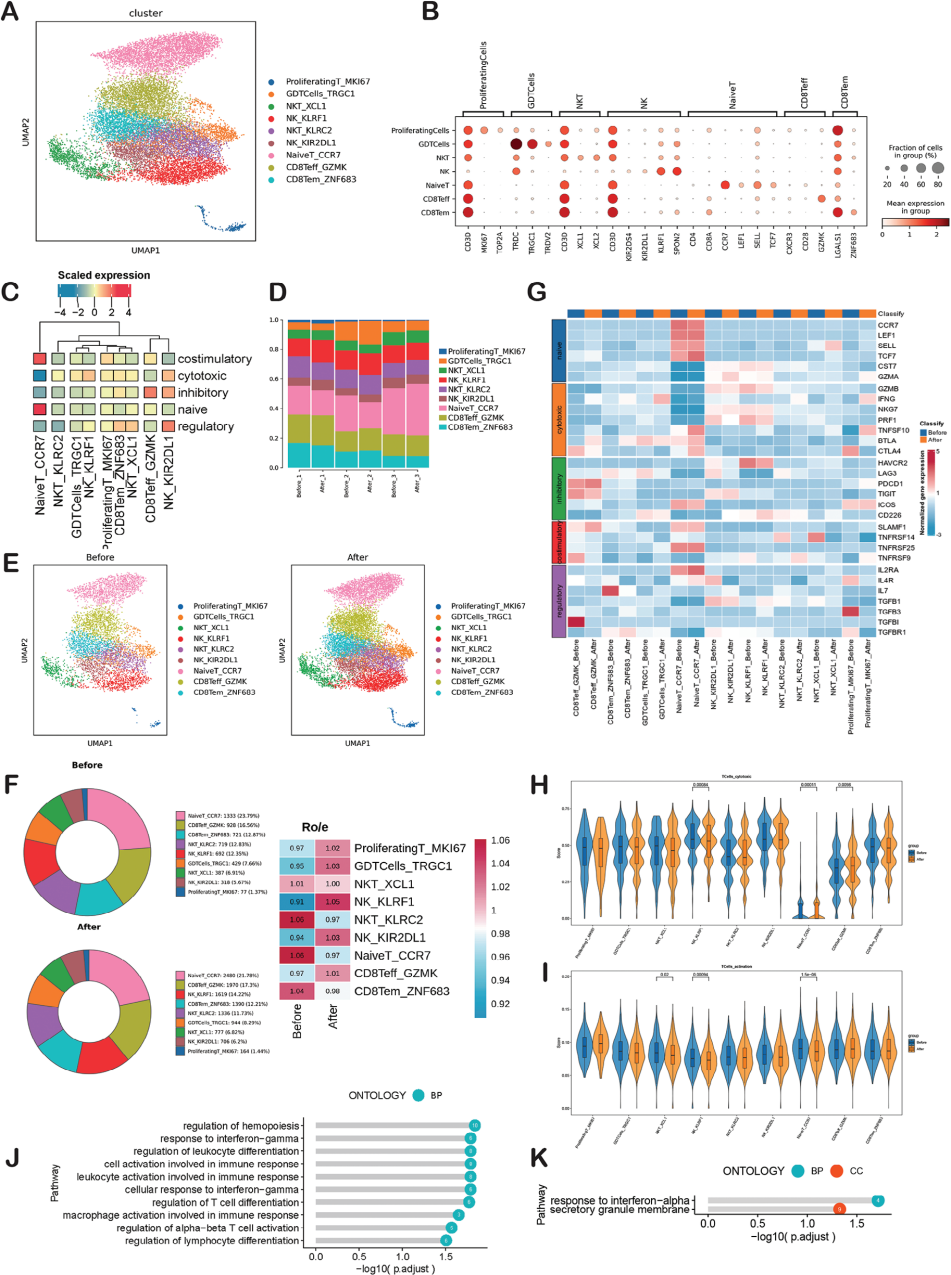
An Overview of T Cells and NK Cells in the Blood. A) UMAP analysis of peripheral T cells and NK cells showing 9 clusters before and after IORT combined with surgery. B) The DotPlot showing the top three signature genes in T cells and NK cells clusters. C) The heat map showing different T cells and NK cells clusters carried out different functions (costimulatory, cytotoxic, inhibitory, naive, and regulatory). D) Histogram indicating the proportion of T cells and NK cells of each sample. E) UMAP plots showing changes of peripheral T cells and NK cells induced IORT+Surgery of breast cancer. F) Pie charts showing the proportions of each T cells and NK cells cluster before and after IORT combined with surgery. Ratio of observed cell number to expected cell number revealed by Ro/e. G) The heatmap illustrates the grouped expression patterns of T cell function‐related genes. H) The cytotoxic scores and I) T cell activation score of different peripheral T cells and NK cells clusters before and after IORT combined with surgery. J) GO enrichment pathway analysis of genes preferentially upregulated in CD8Teff_GZMK cells and K) NK_KLRF1 cells.

Our clustering analysis sub‐grouped T cells and NK cells into 9 clusters (including 7 T cell clusters and 2 NK cell clusters, **Figure** **2**A) based on canonical markers (Figure 2B). T cells including CD8Teff_GZMK, CD8Tem_ZNF683, GDTCells_TRGC1, Naïve T_CCR7, NKT_KLRC2, NKT_XCL1 and proliferating T_MKI67. NK cells including NK_KLRF1 and NK_KIR2DL1. NK_KLRF1 cells specifically expressed KLRF1, GZMB, SPON2, FCER1G, indicative of high activity of cytotoxicity. NK_ KIR2DL1 cells up‐regulated killer cell immunoglobulin receptors (KIR): KIR2DS4, KIR2DL1, KIR2DL3, KLRC2. UCell is an R package specifically designed for assessing gene signature enrichment in scRNA‐seq data. We applied the UCell and found that different cell clusters carried out different functions (costimulatory, cytotoxic, inhibitory, naive and regulatory) in the heat map (Figure 2C). The histogram depicting the general distribution of 9 clusters of T cells and NK cells in each sample (Figure 2D). After IORT, patients exhibited an increased proportion of CD8Teff_GZMK, NK_KLRF1, and NK_KIR2DL1, alongside a decreased proportion of Naïve T_CCR7 in peripheral blood when compared to before IORT levels. (Figure 2E,F). The selected gene expression profiles of various functions within these cell clusters, both before and after IORT+Surgery, were displayed (Figure 2G). Following IORT+Surgery, the expression of the cytotoxic gene TNFSF10 and the regulatory gene IL2RA was enhanced in NaiveT_CCR7 cells. Simultaneously, the regulatory gene TGFBI exhibited reduced expression in CD8Teff_GZMK cells, while IL7R displayed decreased expression in CD8Tem_ZNF683 cells following IORT+Surgery. Meanwhile, CD4 T cells are primarily present in the Naïve T subset. We have analyzed our data to distinguish between CD4 and CD8 within Naïve T cells. We have indeed analyzed the CD4+ T cell subpopulation, specifically focusing on CD4 Naïve T_CCR7 (Figure S1, Supporting Information). Next, we compared the functional scores of each subgroup of T cells between after IORT and before IORT groups. The cytotoxic score of CD8Teff_GZMK cells significantly increased after IORT (Figure 2H). The T cell activation score of NKT_XCL1, NK_KLRF1 and Naïve T_CCR7 cells significantly decreased after radiotherapy (Figure 2I). The costimulatory, naïve, exhaustion, inhibitory and regulatory score were shown in Figure S2 (Supporting Information). The detail of differential gene expression between after IORT and before IORT in 9 subsets were shown in Figure S3 (Supporting Information). In comparison to the state before IORT, gene ontology (GO) enrichment analysis revealed that CD8Teff_GZMK cells after IORT+Surgery exhibited specific enrichment in genes related to several functions, including the regulation of hemopoiesis, cellular response to interferon‐gamma, leukocyte activation involved in immune response, cell activation involved in immune response, regulation of leukocyte differentiation, response to interferon‐gamma, regulation of T cell differentiation, macrophage activation involved in immune response, regulation of alpha‐beta T cell activation, and regulation of lymphocyte differentiation pathway (Figure 2J). Additionally, NK_KLRF1 cells after IORT+Surgery exhibited specific enrichment in genes associated with the response to interferon‐alpha and the secretory granule membrane pathway (Figure 2K).

### 2.3 Clonal Peripheral T‐Cell Expansion after IORT

**Figure 3:**
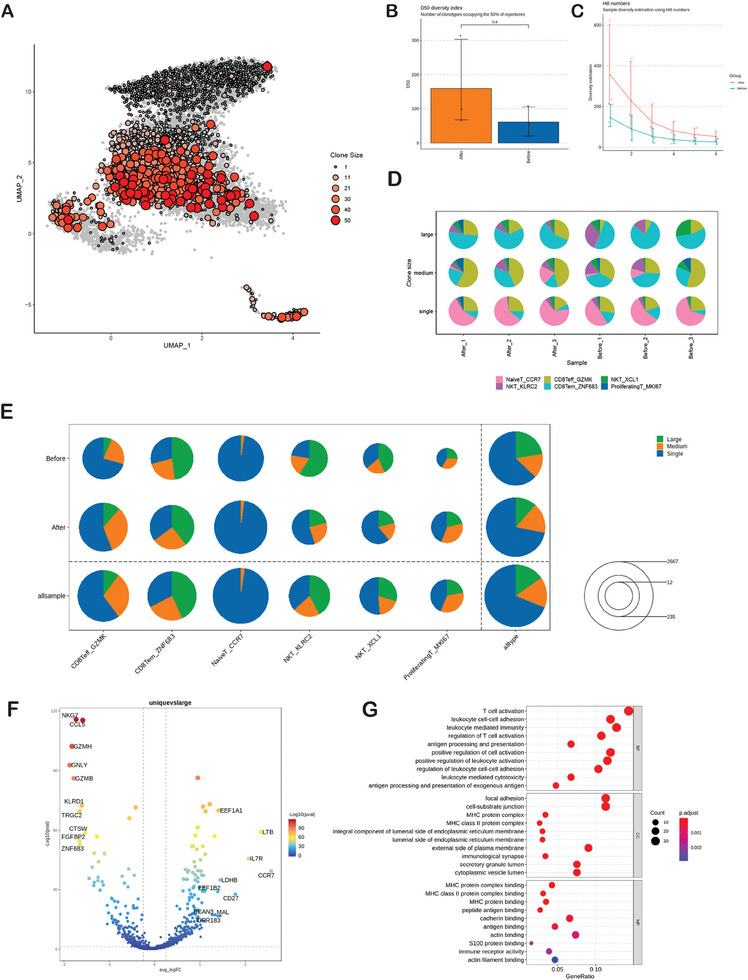
T‐cell clone expansion and TCR repertoire diversity. A) UMAP plot showing the distribution of clonally expanded T cells. The color and size of the dot both reflect the clone size in each cluster. B) TCR diversity was quantified using D50, which resilience with respect to sequencing library size variations. The corresponding p‐value was calculated employing a two‐sided Wilcoxon test. C) Sample diversity estimation using Hill numbers. D) Clonal composition of T cells in samples. The pie charts showing the cell type composition of clonotypes from each sample stratified by clone size (Single:clone size = 1; Medium:1<clone size≤10;Large:clone size>10). E) The percentage of T cells clone size in the blood of after IORT group compared with before IORT group. The size of the circle represents the total number of clones. F) The volcano plot for differentially genes expression (unique vs large clone size). G) GO enrichment analysis showing upregulated pathways in large clone size.

IORT could potentially generate a source of tumor antigen. It is possible that the diversity of peripheral TCR might increase after IORT for breast cancer. To characterize the repertoires of T cell receptors, we performed comparison analysis for Single‐Cell T Cell Receptor Sequencing (scTCR‐seq) data from both before and after IORT+Surgery. The abundance alteration of every individual T‐cell clone in peripheral blood was computed, and the distribution of clonally expanded T cells was depicted through a UMAP plot (**Figure** **3**A). After IORT+Surgery, T cell diversity in peripheral blood appeared to be higher than that observed before the treatment (Figure 3B). Due to the small sample size, the disparity did not achieve statistical significance. We also used Hill numbers for the estimation of sample diversity (Figure 3C). Figure 3D displays the distribution of T cell composition based on clone size in each blood sample. In every cluster, there was a widespread distribution of clonally expanded T cells, especially among CD8Teff_GZMK cells (Figure 3D,E). Moreover, the percentage of T cells with medium or large clone sizes increased in the group after IORT+Surgery as compared to the group before the treatment (Figure 3 E). T cells with large clone size strongly expressed GZMH, GZMB, GNLY, NKG7, related to cytotoxic function (Figure 3F). And T cells with large clone size were specifically enriched in T cell activation, leukocyte mediated immunity, focal adhesion, MHC class II protein complex and immune receptor activity (Figure 3G).

### 2.4 Activated B Cells Induced by IORT of Breast Cancer

**Figure 4:**
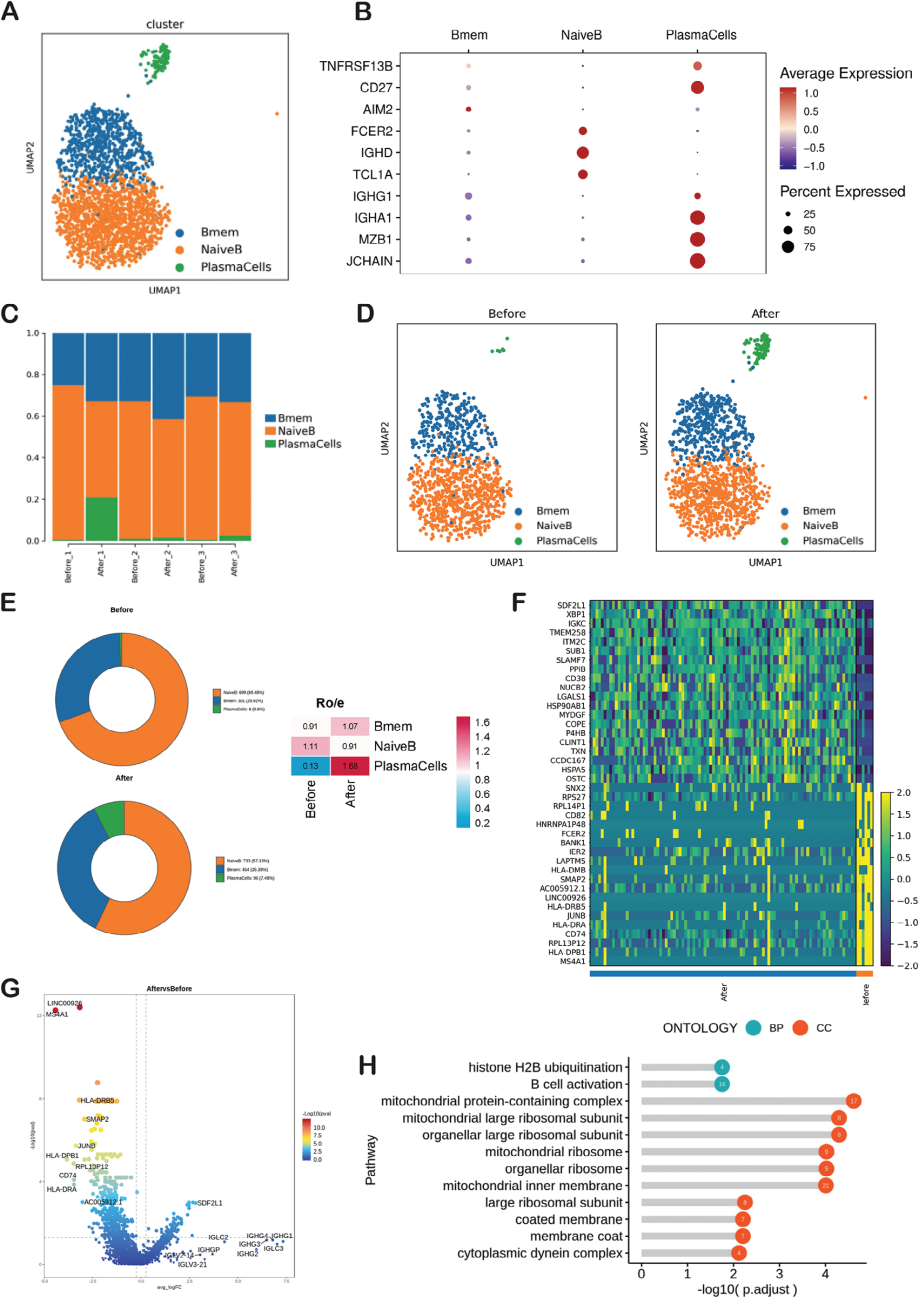
B cells after IORT of breast cancer. A) UMAP plot showing B cell clusters (Naive B cells and Memory B cells) and Plasma cells. B) The DotPlot showing the marker genes in Naive B cells, Memory B cells and Plasma cells. C) The histogram depicting the general distribution of Naive B cells, Memory B cells and Plasma cells in each sample. D) UMAP plots showing changes of peripheral Naive B cells, Memory B cells and Plasma cells induced IORT of breast cancer. E) Pie charts showing the proportions of Naive B cells, Memory B cells and Plasma cells before and after IORT combined with surgery. Ratio of observed cell number to expected cell number revealed by Ro/e. F) The heatmap for differentially genes expression (before IORT vs after IORT). G) The volcano plot for differentially genes expression in Plasma cells (before IORT vs after IORT). H) GO enrichment analysis showing upregulated pathways in Memory B cells after IORT.

We identified two B cell clusters (Naive B cells and Memory B cells) and Plasma cells using scRNA‐seq (**Figure** **4**A). The DotPlot showed the marker genes in Naive B cells, Memory B cells and Plasma cells (Figure 4B). The histogram depicted the general distribution of Naive B cells and Memory B cells and Plasma cells in each sample (Figure 4C). After IORT+Surgery, patients exhibited an increased proportion of Memory B cells and Plasma cells, accompanied by a decreased proportion of Naive B cells in peripheral blood, as compared to the state before IORT (Figure 4D,E). The heatmap depicted the differential gene expression before IORT and after IORT+Surgery (Figure 4F). The results of the differential gene expression analysis revealed that some immunoglobulin genes, such as IGLC2, IGHC3, IGHG1, IGHG2, IGHG3, and IGHG4, tended to be up‐regulated in Plasma cells after IORT+Surgery, but the variation did not reach statistical significance (Figure 4G). The GO enrichment analysis unveiled a significant enrichment of genes associated with histone H2B ubiquitination and B cell activation within the Memory B cell clusters post‐IORT (Figure 4H). Specifically, following IORT+Surgery, genes including LYN, SLC25A5, IGHE, NBN, FCRL3, EXOSC6, IGKC, CEBPG, SAMSN1, IL27RA, SPI1, MZB1, PLCL2, RNF8, VAV3, and GON4L exhibited heightened expression in Memory B cells, indicating an augmented state of B cell activation (Figure 4H).

### 2.5 IORT Increased the Antigen Presenting Function of Conventional DCs Cells

**Figure 5:**
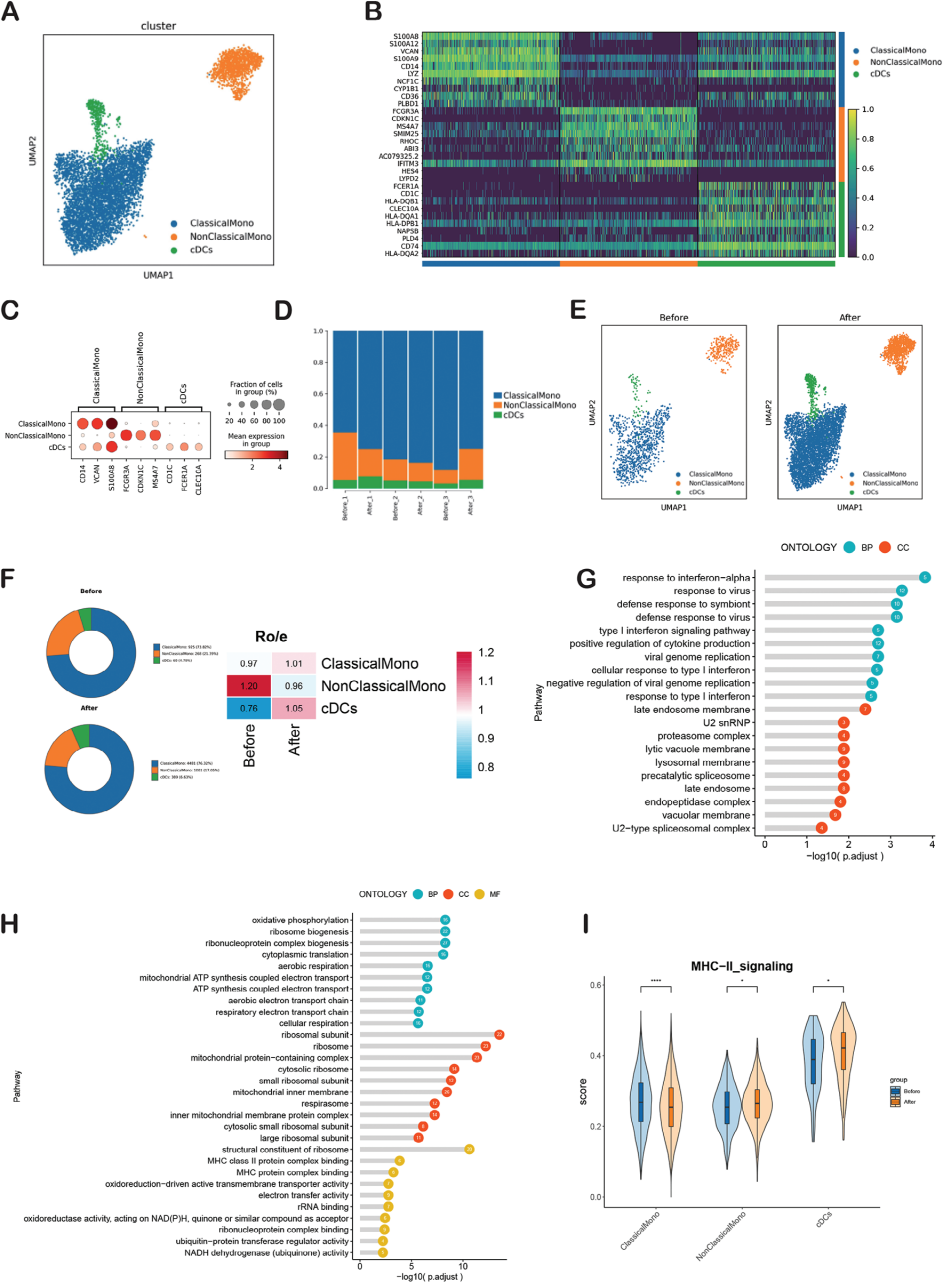
Characterization of MPs responses. A) UMAP plot showing classical monocyte clusters (ClassicalMono), nonclassical monocyte clusters (NonClassicalMono) and Conventional dendritic cells (cDCs). B)The heatmap for differential genes expression (before IORT vs after IORT) in MPs clusters. C) The DotPlot showing the marker genes. D) The histogram depicting the general distribution of ClassicalMono, NonClassicalMono and cDCs in each sample. E) UMAP plots showing changes of peripheral MPs clusters induced IORT of breast cancer. F) Pie charts showing the proportions of ClassicalMono, NonClassicalMono and cDCs before and after IORT combined with surgery. Ratio of observed cell number to expected cell number revealed by Ro/e. GO enrichment analysis showing upregulated pathways in G) NonClassicalMono cells and H) cDCs cells after IORT combined with surgery. I) The UCell Score results of antigen presenting function in cDCs cells.

IORT performed in situ could generate an antigen source for the development of antitumor immunity. Antigen‐Presenting Cells (APCs), particularly professional APCs like dendritic cells (DCs) and macrophages, present these antigens to T cells. To ascertain the specific APCs involved in the immune response induced by IORT+Surgery, we examined changes in the transcriptional profiles of peripheral monocytes and DCs. Through re‐clustering mononuclear phagocytes cells, we distinguished three discrete cell types based on canonical markers: classical monocyte clusters (ClassicalMono), nonclassical monocyte clusters (NonClassicalMono), and Conventional dendritic cells (cDCs) (**Figure** **5**A–C). The histogram depicting the general distribution of ClassicalMono, NonClassicalMono and cDCs in each sample (Figure 5D). Compared with that before IORT, ClassicalMono and cDCs showed a slight increase proportion, and NonClassicalMono showed a decrease proportion after IORT+Surgery (Figure 5E,F). In comparison to the state before IORT, GO enrichment analysis revealed that the NonClassicalMono clusters after IORT+Surgery were notably enriched in genes related to several functions, including the response to interferon‐alpha, positive regulation of cytokine production, type I interferon signaling pathway, cellular response to type I interferon, response to type I interferon, cytokine‐mediated signaling pathway, T cell activation and positive regulation of leukocyte mediated immunity (Figure 5G). Meanwhile, cDCs after IORT+Surgery were specifically enriched in genes associated with MHC class II protein complex binding and MHC protein complex binding signaling pathway (Figure 5H). And the UCell Score results also indicated an upregulation of the MCH II signaling pathway in cDCs cells after IORT (Figure 5I). These results indicated that IORT+Surgery can increase the antigen presenting function of cDCs cells.

### 2.6 IORT Inhibited the Function of Neutrophils

**Figure 6:**
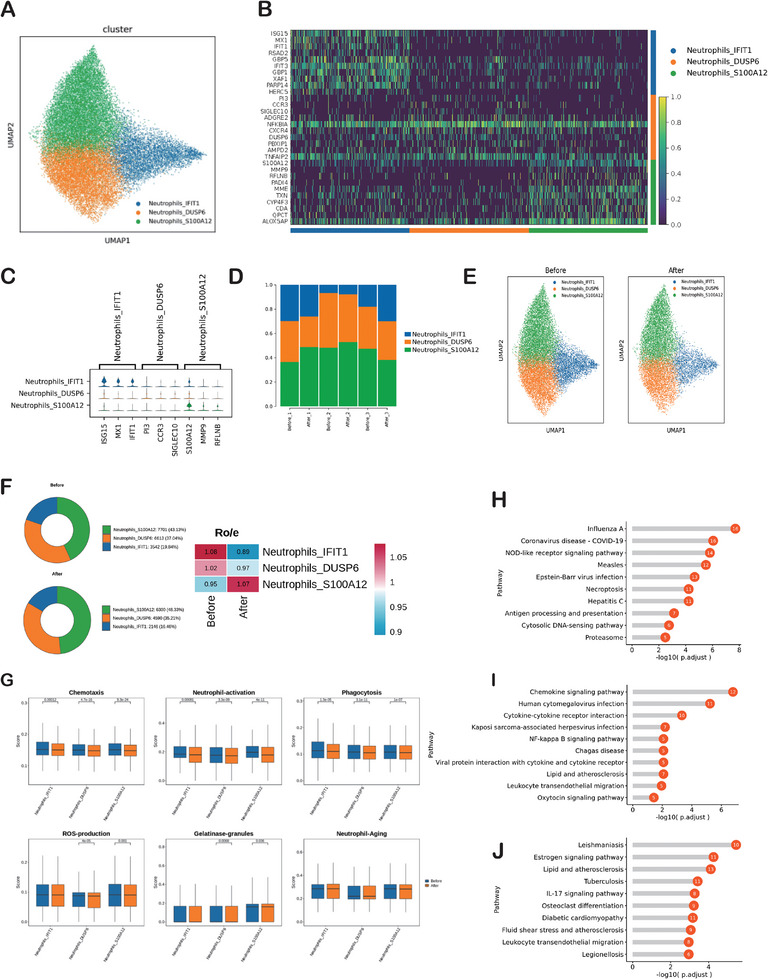
Characterization of Neutrophils. A) UMAP plot showing three neutrophils clusters: Neutrophils_IFIT1 cells; Neutrophils_DUSP6 cells and Neutrophils_S100A1 cells. B) The heatmap for differential genes expression (before IORT vs after IORT) in neutrophils clusters. C) The marker genes of Neutrophils_IFIT1 cells; Neutrophils_DUSP6 cells and Neutrophils_S100A1 cells. D)The histogram depicting the general distribution of Neutrophils_IFIT1 cells, Neutrophils_DUSP6 cells and Neutrophils_S100A12 cells in each sample. E)UMAP plots showing changes of peripheral Neutrophils clusters induced by IORT of breast cancer. F) Pie charts showing the proportions of Neutrophils_IFIT1 cells, Neutrophils_DUSP6 cells and Neutrophils_S100A12 cells before and after IORT+Surgery. Ratio of observed cell number to expected cell number revealed by Ro/e. G) The chemotaxis, neutrophil‐activation, phagocytosis, ROS‐production, gelatinase‐granules and neutrophil‐aging scores. KEGG analysis showing upregulated pathways in H) Neutrophils_IFIT1 cells, I) Neutrophils_DUSP6 cells and J) Neutrophils_S100A12 cells after IORT combined with surgery.

We identified three neutrophils’ clusters using scRNA‐seq: Neutrophils_IFIT1 cells; Neutrophils_DUSP6 cells and Neutrophils_S100A1 cells (**Figure** **6**A). The heatmap for differential genes expression (before IORT+Surgery versus after IORT+Surgery) in neutrophils clusters was shown in Figure 6B. Neutrophils_IFIT1 cells expressed ISG15, MX1 and IFIT1; Neutrophils_DUSP6 cells expressed PI3, CCR3 and SIGLEC10; Neutrophils_S100A12 cells expressed S100A12, MMP9 and RFLNB (Figure 6C). The functions of different neutrophils clusters were showed in Figure S4 (Supporting Information). The histogram depicting the general distribution of Neutrophils_IFIT1 cells, Neutrophils_DUSP6 cells and Neutrophils_S100A12 cells in each sample (Figure 6D). The total number of neutrophils decreased after IORT+Surgery, both Neutrophils_IFIT1 cells and Neutrophils_DUSP6 cells showed a tendency to decrease (Figure 6E,F). Then, we further evaluated the functional changes of each neutrophil's clusters before and after IORT+Surgery. After IORT+Surgery, all the neutrophils’ clusters showed chemotaxis, neutrophil‐activation and phagocytosis scores significant decreased (Figure 6G). The ROS‐production scores and gelatinase‐granules scores also decreased in Neutrophils_DUSP6 cells and Neutrophils_S100A12 cells after IORT+Surgery (Figure 6G). IORT+Surgery had no significant effect on the neutrophil‐aging scores (Figure 6G). According to KEGG analysis, Neutrophils_IFIT1 cells after IORT+Surgery exhibited specific enrichment in genes associated with pathways such as the NOD‐like receptor signaling pathway, Necroptosis, Antigen processing and presentation, and NF‐kappa B signaling pathway (Figure 6H). Neutrophils_DUSP6 cells after IORT+Surgery were particularly enriched in genes related to the Chemokine signaling pathway, Cytokine‐cytokine receptor interaction, NF‐kappa B signaling pathway, Leukocyte transendothelial migration, Oxytocin signaling pathway, IL‐17 signaling pathway, Necroptosis, Th17 cell differentiation, and NOD‐like receptor signaling pathway (Figure 6I). Neutrophils_S100A12 cells after IORT+Surgery exhibited specific enrichment in genes associated with the IL‐17 signaling pathway, Neutrophil extracellular trap formation, NOD‐like receptor signaling pathway, Platelet activation, PD‐L1 expression and PD‐1 checkpoint pathway in cancer, Apoptosis, Chemokine signaling pathway, Th17 cell differentiation, HIF‐1 signaling pathway, and TNF signaling pathway (Figure 6J).

### 2.7 Cell‐To‐Cell Communication Among Immune Cells

**Figure 7:**
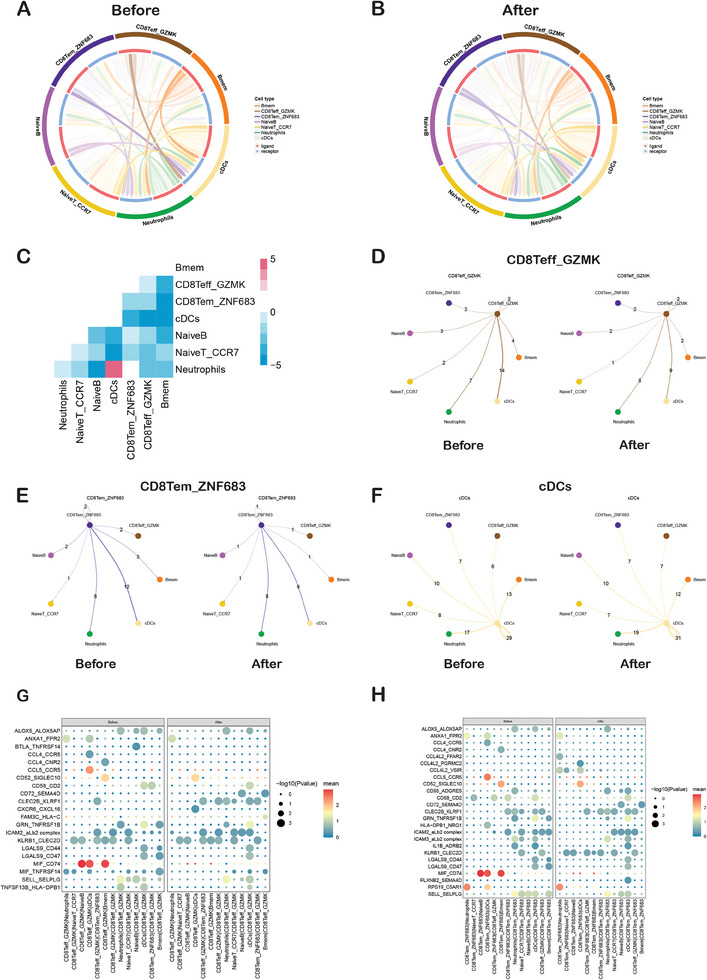
Cell‐to‐cell communications. Analysis was performed using CellPhoneDB. This diagram illustrates the quantity of interactions between two cell types A) in Before IORT group or B) After IORT group. Outer ring color blocks represent cell types; inner ring red represents ligands, and blue represents receptors. The clarity of the lines is positively correlated with the quantity of interactions between the two cell types. C) The heatmap shows the difference in the number of interacting pairs between two groups (After IORT group – Before IORT group). Red indicates more interacting pairs in After IORT group, while blue indicates more interacting pairs in Before IORT group. D–F) A detailed view of ligands expressed by each major cell type before and after IORT combined with surgery. G) Overview of selected ligand‐receptor interactions between CD8Teff_GZMK cells and other immune cells before and after IORT+Surgery. H) Overview of selected ligand‐receptor interactions between CD8Tem_ZNF683 cells and other immune cells before and after IORT+Surgery. X‐axis: Cell Type Pairs, “ligand cell | receptor cell”; Y‐axis: Interacting Gene Pairs, “ligand gene_ receptor gene”.

CellphoneDB was utilized to forecast cell‐cell interactions that could play a role in the immune response triggered by IORT+Surgery. We assessed these interactions among various cell types both before and after IORT+Surgery (**Figure** **7**A,B). Interestingly, the majority of cell interaction pairs decrease after IORT+Surgery, except for the increased interaction pairs between cDCs and Neutrophils (Figure 7C). We found reduced interactions between cDCs cells and CD8Teff_GZMK, CD8Tem_ZNF683, NaiveT_CCR7, Naïve B or Memory B cells in the peripheral blood after IORT+Surgery in comparison to that before IORT (Figure 7C). A detailed view of ligands expressed by each major cell type before and after IORT+Surgery were shown in Figures 7D–F and S5 (Supporting Information). These findings suggest that a new balance emerged after IORT+Surgery. Next, analysis of secreted ligands for the detected cognate receptors. Communication between CD8Teff_GZMK cells and other immune cells via MIF_CD74 and MIF_TNFRSF14 was decreased after IORT (Figure 7G). We also found CD8Tem_ZNF683 and other immune cells via MIF_CD74 was decreased after IORT+Surgery (Figure 7H). DCs stand as the most effective professional antigen‐presenting cells, displaying antigens via MHC class I and II molecules to both CD8+ and CD4+ T cells. Here, enhanced CXCR6_CXCL16 and CD52_SIGLEC10 between CD8Teff_GZMK and cDCs cells were observed after IORT+Surgery (Figure 7G). Moreover, enhanced interactions with CCL4L2_PGRMC2, CCL4L2_VSIR, CD52_SIGLEC10 between CD8Tem_ZNF683 cells and DCs were found after IORT+Surgery (Figure 7H).

### 2.8 IORT Boosts CD8+ T Cell Activity and Synergy with PD‐1 Blockade

**Figure 8:**
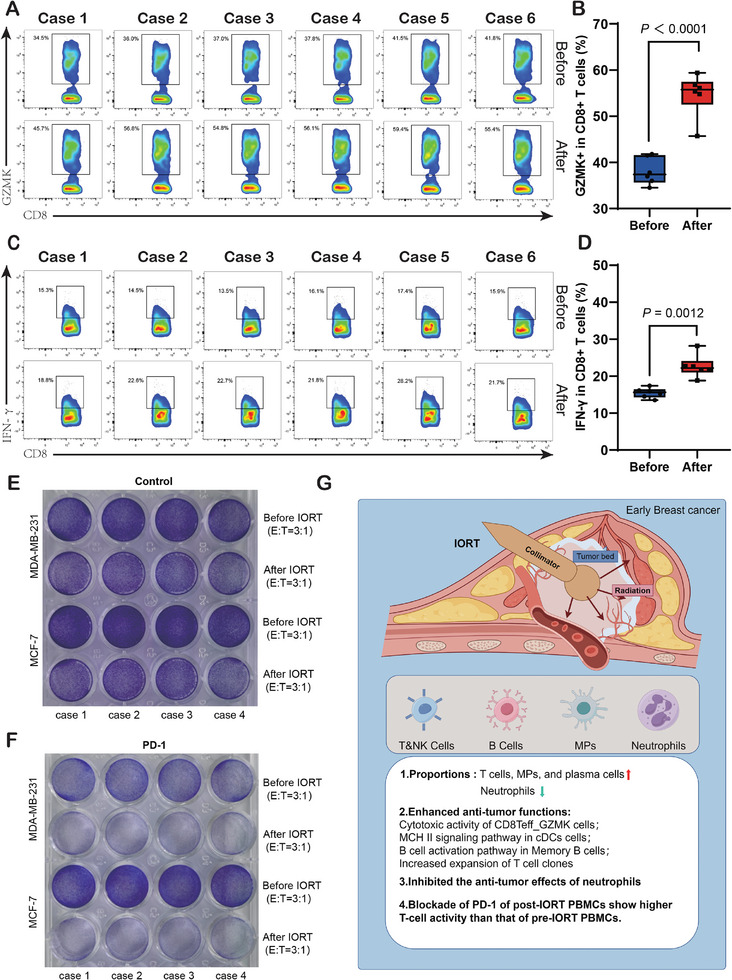
Validations of IORT induced immune response and combination experiments in vitro. Representative flow cytometry images and the statistical data of A,B) granzyme B+ CD8+ T cells and C,D) IFN+ CD8+ T cells of PBMCs in six breast cancer patients who received IORT combined with surgery. E,F) T cell‐mediated cancer cell‐killing assay results. MDA‐MB‐231 or MCF‐7 cells co‐cultured with T cells at an E:T ratio of 3:1 upon the addition of without or with 10 µg ml^−1^ pembrolizumab (PD‐1 inhibitor) for 48 h, followed by crystal violet staining. G) Major findings in graphical form.

PBMCs were collected from six breast cancer patients before and after IORT+Surgery for subsequent experiments. Flow cytometry analysis was performed to assess the frequency of GZMK+ cells in CD8+ T cells (**Figure** **8**A,B) and IFN‐γ+ cells in CD8+ T cells (Figure 8C,D) isolated from PBMCs. The results revealed a significant increase in the proportion of GZMK+ cells and IFN‐γ+ cells in CD8+ T cells after IORT+Surgery (GZMK+ P <0.0001; IFN‐γ+ P = 0.0012). These findings suggest an enhancement in the cytotoxicity of CD8+ T cells following IORT+Surgery. PD‐1, as a co‐inhibitory molecule, plays a pivotal role in T cell function. Blocking PD‐1 leads to the restoration of T cell function and promotes effective anti‐tumor responses. MDA‐MB‐231 or MCF‐7 cells were co‐cultured with activated T cells at an E:T ratio of 3:1, without or with 10 µg/ml pembrolizumab (PD‐1 inhibitor), for 48 h, followed by crystal violet staining. We found that IORT+Surgery enhanced human T cell‐mediated cytotoxicity (Figure 8E). Additionally, PBMCs cultured with pembrolizumab after IORT+Surgery exhibited increased human T cell‐mediated cytotoxicity compared to PBMCs collected before IORT (Figure 8F). These findings indicate that peripheral T cells exposed to IORT+Surgery treatment are synergistically activated when PD‐1 is blocked.

## 3 Discussion

Radiation therapy constitutes a primary treatment approach for solid tumors and serves as a regulator of the immune response against these tumors.^[^ ^18^ ^]^ There were no previous investigations into the influence of IORT on peripheral immune cells at the single‐cell level. We collected peripheral blood samples from three breast cancer patients at two time points: before intraoperative radiotherapy and one week after intraoperative radiotherapy combined with surgery. Peripheral blood mononuclear cells were isolated and subjected to scRNA‐seq and scTCR‐seq analysis to examine characteristic changes in PBMCs. Based on the results of this study, in early‐stage breast cancer patients undergoing breast‐conserving surgery, the combination of IORT treatment and surgery can influence the equilibrium of immune cells in peripheral blood.

From a radiobiological point of view, radiation therapy can cause lymphopenia because lymphocytes are extremely sensitive to radiation.^[^ ^19^ ^]^ Circulating lymphocytes are 70%‐80% composed of T lymphocytes.^[^ ^20^ ^]^ T cells can be differentiated to different sub‐groups with different immunocompetences. Different T cell sub‐groups have different responses to radiotherapy in different cancers. For example, Yao et al. found that memory T cells are considerably more resistant to radiation than naïve T cells.^[^ ^21^ ^]^ CD4+ T cells exhibit greater radiosensitivity than CD8+ T cells and regulatory T cells.^[^ ^22^, ^23^ ^]^ Subgroup analysis of different cancer types showed that in head and neck cancer, T lymphocytes decreased after radiotherapy. However, there was no notable alteration in peripheral blood T lymphocytes after radiotherapy in prostate cancer. Meanwhile, radiotherapy holds the potential to stimulate the proliferation and activation of lymphocytes in lung cancer. These variations are intricately tied to the primary tumor type, radiation techniques, dosage regimen, and timing of blood sample collection.^[^ ^24^ ^]^ In this study, we observed that while the absolute count of PBMCs remained relatively stable after IORT+Surgery, there was an elevation in the proportions of T cells, MPs and plasma cells. This shift in composition was concurrent with a reduction in the proportion of neutrophils. Functionally, we found that the cytotoxic score of CD8Teff_GZMK cells significantly increased after IORT. And the CD8Teff_GZMK cells upregulates cellular response to interferon‐gamma, immune response and T cell differentiation pathways. The increase in GZMK+ and IFN‐γ+ CD8+ T cells post‐IORT suggests a potentiation of the cytotoxic capabilities of these cells. Granzyme K, a serine protease, plays a crucial role in the direct killing of target cells through the induction of apoptosis. Meanwhile, IFN‐γ is a critical cytokine for antitumor immunity, enhancing the antigen presentation process and exerting direct antiproliferative effects on tumor cells.^[^ ^25^ ^]^ Therefore, the observed increase in these markers indicates an active cytotoxic immune response post‐IORT, which is likely contributing to tumor control and the reduction of recurrence risk. The expansion of T‐cell clones was validated through TCR sequencing. Clonally expanded T cells were found to be prevalent across various T cell clusters, with a notable concentration in CD8Teff_GZMK cells. Meanwhile, we observed that T cell diversity tended to be higher in the blood after IORT compared to before IORT. The TCR diversity may reflect the probability of neoantigen recognition.^[^ ^26^, ^27^ ^]^ Recent studies further support the significance of these findings. Linares‐Galiana et al. found that IORT in breast cancer patients led to significant immunostimulatory effects, potentially enhancing antitumor immunity by increasing NK cell counts and possibly affecting T cell diversity.^[^ ^12^ ^]^ Additionally, Wuhrer et al. discussed the altered cytokine profiles in wound fluid from breast cancer patients undergoing IORT, indicating potential modifications in the immune response that could influence T cell behavior.^[^ ^28^ ^]^

Immunotherapy has brought about a revolution in cancer treatments in the past few years.^[^ ^29^ ^]^ Previous studies have explored the combined effects of local treatments (such as microwave ablation) and immune checkpoint inhibitors in cancer. For example, Zhou et al. observed that in breast cancer, immune checkpoint inhibitors had a synergistic activation effect on peripheral T cells following microwave ablation.^[^ ^30^ ^]^ Currently, there is no exploration of the combined use of intraoperative radiotherapy and immune checkpoint inhibitors in breast cancer. In vitro experimental, we focused on validating the functional impact of IORT on T cells and further explored the influence of these T cells on immunotherapy. Previous studies have found a correlation between pre‐treatment tumor‐infiltrating T lymphocytes and PD‐1 blockade response, with immune‐infiltrated tumors generally exhibiting better responses compared to immune‐excluded tumors.^[^ ^31^ ^]^ T‐cell kinetic studies have indicated that the response of T cells to immune checkpoint blockade may originate from outside the tumor and depend on the recruitment of peripheral T cells.^[^ ^32^ ^]^ Our experimental results have confirmed that after IORT+Surgery, the activation of peripheral blood T‐cell function is enhanced, and when combined with PD‐1 inhibitors, it can augment the tumor‐killing function. Han et al. explored the combination of a PI3Kγδ inhibitor with radiation and PD‐1 blockade in a murine breast cancer model.^[^ ^33^ ^]^ Their findings suggest that this tripartite approach not only delays primary tumor growth but also enhances the abscopal effect, thereby improving overall survival. This indicates a significant synergistic potential when integrating PD‐1 blockade with radiation, which could be pivotal in treating immunologically cold tumors such as breast cancer.

In addition to the increased expansion of T cell clones and enhanced anti‐tumor capabilities of T cells, we also observed that intraoperative radiotherapy combined with surgery enhances some anti‐tumor functions of other immune cells. For instance, there was an upregulation of the MCH II signaling pathway in cDCs cells which increased in the antigen presentation capacity of cDCs cells. MHC class II molecules are crucial for presenting antigens to CD4+ T cells, initiating adaptive immune responses.^[^ ^34^ ^]^ Radiation can kill cancer cells and release tumor antigens.^[^ ^35^ ^]^ Lhuillier et al. explored the effects of radiotherapy on the upregulation of MHC class II molecules and demonstrated that radiotherapy‐exposed neoantigens on CD4+ T cells improved tumor control, which supports the notion of enhanced antigen presentation capacity.^[^ ^36^ ^]^ Upon exposure to antigens, cDCs process and present antigenic peptides bound to MHC class II molecules on their cell surface.^[^ ^37^ ^]^ The upregulation of the MHC protein complex binding signaling pathway in cDCs indicates increased intracellular signaling events associated with antigen presentation. This signaling pathway likely involves various molecular interactions and signaling cascades that optimize antigen processing, MHC molecule trafficking, and antigen presentation efficiency.

Meanwhile, we also found the increase in the proportions of memory B cells and plasma cells and the decrease in the proportion of naive B cells in peripheral blood after IORT+Surgery. Memory B cells after IORT+Surgery enhanced B cell activation pathway. The increase in memory B cells and plasma cells suggests a heightened adaptive immune response.^[^ ^38^ ^]^ Memory B cells are critical for rapid and robust antibody production upon re‐exposure to antigens, implying enhanced readiness against tumor antigens that may recur post‐treatment. Increased plasma cells indicate active antibody production which could contribute to ongoing immune surveillance and response against residual cancer cells. A decrease in naive B cells may indicate their differentiation into memory and plasma cells, a sign of an evolving adaptive immune response.^[^ ^39^ ^]^ This shift could reflect the immune system's adaptation to the presence of tumor antigens introduced by IORT+Surgery, potentially improving the specificity and efficiency of the immune response against breast cancer cells. The modulation of B cell subsets after IORT+Surgery could be an important factor in the long‐term immune response against breast cancer, affecting both the efficacy of the initial treatment and long‐term surveillance. These findings could inform the development and timing of immunotherapeutic strategies post‐IORT. For example, treatments that enhance the efficacy of memory B cells or support plasma cell functions could be beneficial in prolonging survive in breast cancer patients.^[^ ^38^, ^40^ ^]^ Our findings support the notion that IORT serves not only as an efficacious local treatment but also as a stimulator of antitumor immunity in early‐stage breast cancer.

Neutrophils are the most abundant white blood cells and play dual roles in cancer advancement.^[^ ^41^ ^]^ This versatile behavior is likely a result of their unforeseen adaptability in reacting to environmental signals. Despite their cytotoxic capabilities, neutrophils are commonly exploited by malignancies to foster immunosuppression, tumor proliferation, and metastatic progression.^[^ ^42^ ^]^ In most cases, tumor‐associated neutrophils has been correlated with an unfavorable prognosis in human tumors.^[^ ^43^ ^]^ Our results suggest that intraoperative radiotherapy can lead to a decrease in the function of neutrophils, including chemotaxis, phagocytosis and neutrophil‐activation. The ROS‐production scores and gelatinase‐granules scores also decreased in Neutrophils_DUSP6 cells and Neutrophils_S100A12 cells after IORT+Surgery. The most well‐known example of neutrophil‐directed cytotoxicity against tumor cells is the production of reactive oxygen species. Neutrophils possessing antitumor characteristics are capable of engaging in direct tumor cell destruction through the release of reactive oxygen species and reactive nitrogen species.^[^ ^44^ ^]^ Conversely, pro‐tumor neutrophils can release MMP9, promoting tumor cell angiogenesis and dissemination.^[^ ^45^ ^]^ In our study, we observed that Neutrophils_S100A12 cells were specifically enriched in genes associated with IL‐17 signaling pathway after IORT+Surgery, which including MMP9/ S100A8/ S100A9/ IL17RA/ JUN/ FOS/ MAPK14/ CXCL1 genes. Our results have demonstrated that intraoperative radiotherapy combined with surgery can, on one hand, induce damage to neutrophils (resulting in a reduction in their numbers), and on the other hand, to some extent, inhibit the anti‐tumor effects of neutrophils.

In cell‐cell communication, we observed that the majority of cell interaction pairs decrease after IORT+Surgery, except for the increased interaction pairs between cDCs and Neutrophils. The MIF_CD74 axis is downregulated among multiple cell types. Some research findings have indicated that the MIF_CD74 axis might inhibit the anti‐tumor immune response by either recruiting tumor‐associated macrophages or directly dampening T cell activation.^[^ ^46^ ^]^ Therefore, our findings support the upregulation of the anti‐tumor immune response after intraoperative radiotherapy. Additionally, we also found enhanced interactions of CXCR6_CXCL16 and CD52_SIGLEC10 between CD8Teff_GZMK and cDCs cells after IORT+Surgery, as well as enhanced interactions of CD52_SIGLEC10 between CD8Tem_ZNF683 cells and DCs after IORT+Surgery. CXCR6‐CXCL16 could provide survival signals for T cells.^[^ ^47^ ^]^ Previous studies indicates that the interaction between cDCs and CD8+ T cells is fundamental for the activation of CD8+ T cells and subsequent cytotoxic responses, which are essential for the immune surveillance against tumors.^[^ ^48^, ^49^ ^]^ CD52 is a significant immunomodulatory factor for T cell activation. It can regulate T cell activation through its intracellular signaling pathways or by interacting with Siglec‐10.^[^ ^50^ ^]^ Therefore, our results suggest that the activity of CD8Teff_GZMK and CD8Tem_ZNF683 may increase after IORT+Surgery. These changes in ligand expression are indicative of a dynamic reprogramming of immune functions following IORT+Surgery, which may be crucial for the subsequent immune surveillance and response to residual tumor cells.

Some limitations persist in this study. First, chemotherapy or endocrine therapy interventions being executed on these patients within two weeks after IORT. Therefore, only short‐term immune responses were gauged, and necessitating the exploration of long‐term immune reactions in forthcoming research endeavors. Second, we did not observe the regulatory T cells, which are crucial in tumor cells evading immune surveillance. Additionally, our T cell assays lacked tumor‐specific markers, which limits our ability to precisely attribute the observed immune responses to specific tumor antigens. Incorporating tumor‐specific markers in future studies would enable a more accurate assessment of how T cells target tumor cells and could provide deeper insights into the effectiveness of the combined IORT and surgical interventions. Third, the restricted sample size confined our findings to emerge in isolation from diverse clinicopathological variables and disparate local controls. Although an apparent increase in T cell diversity in peripheral blood was observed after IORT+Surgery compared to before, these trends did not achieve statistical significance due to the small sample size. Subsequently, extensive studies encompassing larger sample sizes are imperative to discern prospective beneficiaries of this localized therapy. Fourth, the precise anti‐tumor efficacy of combining IORT with immune checkpoint inhibitors remains to be determined. While radiation therapy is known to enhance immune responses through immunogenic cell death, IORT's targeted approach may limit this effect by focusing primarily on the tumor bed with minimal impact on surrounding normal tissue. Consequently, the observed immune activation post‐IORT may not only stem from the immunogenic death of tumor cells but also from an immune recovery phase following tumor resection. Therefore, it is crucial that upcoming clinical trials clarify the effects of this combined strategy. Lastly, the immune responses observed in our study were influenced by the combination of surgery and IORT, not by IORT alone. Future studies should include a control group undergoing surgery alone to differentiate the specific contributions of each intervention.

## 4 Conclusion

In this study, we utilized scRNA‐seq and scTCR‐seq to investigate the impact of intraoperative radiotherapy on peripheral blood immune cells in early breast cancer. We have preliminarily established a dynamic landscape of peripheral blood mononuclear cells associated with intraoperative radiotherapy. Our study help clarify the interactions between IORT and the host immune system in breast cancer patients. Furthermore, we preliminary investigated the effectiveness of combining intraoperative radiotherapy with immunotherapy in vitro experiments. This study characterizes the systemic immune response induced by IORT+Surgery and paves the way for identifying potential targets to enhance immune responses.

## 5 Experimental Section

### Patients

The patients involved in this study were from Guangdong Provincial People's Hospital and the Second Affiliated Hospital of Guangzhou Medical University. The inclusion criteria for this study include: 1) a solitary tumor; 2) confirmation of invasive breast cancer through core‐needle biopsy; and 3) female and 18 years of age or older, with no coagulative disorders, chronic liver disease, renal failure, immune system disorders, or any other acute or chronic conditions that could potentially affect the immune response. Before undergoing surgery, no patient with breast cancer was given neoadjuvant chemotherapy. Approval for the current research was obtained from the Ethics Committee (GDREC2019497H and KY‐Z‐2022‐362‐02).

### Intraoperative Radiotherapy System

For intraoperative radiotherapy, the INTRA‐BEAM system, produced by Carl Zeiss Surgical in Oberkochen, Germany, was utilized. The target tumor bed was exposed to a high dose rate of low‐energy photons (50 kV) emitted by this system, resulting in a rapid reduction in dosage.

### Collection of Peripheral Blood and Isolation of Mononuclear Cells

The peripheral blood was withdrawn on the day before and one week after IORT. Venous blood was collected in EDTA anticoagulant tubes (10 ml) and transported to laboratory under cooled conditions (4–6 °C) for immediate isolation of PBMC. The PBMCs were obtained through a process of density gradient centrifugation utilizing Ficoll‐Paque Plus medium (GE Healthcare) and subsequently rinsed with Ca/Mg‐free PBS. To eliminate red blood cells, a volume of 2 mL of GEXSCOPE red blood cell lysis buffer (RCLB, Singleron) was added and incubated at 25 °C for a duration of 10 min. The solution was then subjected to centrifugation at 500 × g for 5 min and re‐suspended in PBS. Subsequently, the blood samples were subjected to centrifugation at 400 g for 5 min at 4 °C and the supernatant was discarded. After the removal of red blood cells, PBMCs were isolated through centrifugation at 400 g for 10 min at 4 °C. The supernatant was discarded and the PBMCs were re‐suspended in phosphate‐buffered saline to achieve a state of single‐cell suspension. Ultimately, the samples were subjected to staining using Trypan Blue, and the viability of the cells was assessed under a microscope.

### Single‐Cell RNA Sequencing

Cellular viability of all freshly isolated PBMCs exceeded 90%, as assessed microscopically using trypan blue (Sigma, USA). Single‐cell suspensions in PBS were loaded into microfluidic devices using the Singleron Matrix Single Cell Processing System from Singleron Biotechnologies in China. Subsequently, single‐cell RNA sequencing (scRNA‐seq) libraries were constructed following the GEXSCOPE Single Cell RNA Library Kits protocol, also from Singleron Biotechnologies, China. Individual libraries were diluted to a concentration of 4 × 10^−9^ m and pooled for sequencing. These pooled samples were then sequenced on an Illumina HiSeq X platform, generating 150 bp paired‐end reads.

### Construction of scTCR‐seq Libraries

The suspension of individual cells, at a density of 1 × 10^5^ cells ml^−1^, was inserted into the microfluidic apparatus. Afterward, scTCR‐seq libraries were created using the GEXSCOPE Single Cell Immuno‐TCR/BCR Kit procedure provided by Singleron Biotechnologies in China. Briefly, magnetic particles with molecular markers were employed to capture the poly(A) tail and T‐cell receptor (TCR) area on mRNA, marking both cells and mRNA subsequent to cell breakdown. Afterward, the chip collected magnetic beads, which captured mRNAs. These magnetic beads were then used for reverse transcription into complementary DNA (cDNA) and subsequent amplification. After fragmenting and splicing partial cDNA, libraries appropriate for the Illumina sequencing platform were created. The rest of the cDNA was specifically concentrated for the immune receptor (TCR), and the concentrated products were amplified through PCR to create a sequencing library that was compatible with the Illumina sequencing platform. In the end, every library underwent sequencing using the Illumina HiSeq X, resulting in the production of 150 bp paired‐end reads.

### Clonotype Expansion

Following the methodology outlined in previous literature,^[^ ^51^ ^]^ clonotype expansion was defined as follows: 1) an increase in frequency (i.e., the number of cells with the same TCR) or proportion (i.e., frequency normalized to the number of cells in a sample with a detected TCR) after treatment compared to before treatment, and 2) a frequency greater than 2 after treatment.

### Primary Analysis of Raw Read Data of scRNA‐seq

Gene expression profiles were generated by processing raw reads with CeleScope v1.5.2 from Singleron Biotechnologies, using the default settings. To summarize, the barcodes and UMIs were obtained from R1 reads and then rectified. The R2 reads had their adapter sequences and poly A tails removed, and then they were aligned to the GRCh38 (hg38) transcriptome using STAR (v2.6.1b). After employing FeatureCounts (v2.0.1), the reads that were uniquely mapped were allocated to exons. To generate the gene expression matrix, reads that shared the same barcode, UMI, and gene were grouped together.

### Cell‐Type Recognition with Cell‐ID

The categorization of each cluster's cell type was ascertained based on the expression of canonical markers retrieved from the SynEcoSysTM reference database provided by Singleron Biotechnology. SynEcoSysTM amalgamates a compendium of recognized cell type markers curated from diverse sources, including CellMakerDB, PanglaoDB, and recently published scientific literature on single‐cell sequencing data.

### Differentially Expressed Genes (DEGs) Analysis

To identify differentially expressed genes (DEGs), the scanpy.tl.rank_genes_groups function was used, which relies on the Wilcoxon rank sum test using default parameters. Genes expressed in over 10% of cells within each compared cell group, with an average log (Fold Change) value exceeding 1, were designated as DEGs. The adjusted p‐value was computed through Benjamini‐Hochberg correction, and a significance threshold of 0.05 was applied to assess statistical significance.

### Pathway Enrichment Analysis

To explore the potential functions of differentially expressed genes (DEGs), Gene Ontology (GO) and Kyoto Encyclopedia of Genes and Genomes (KEGG) analysis were employed with the “clusterProfiler” R package version 3.16.1.^[^ ^52^ ^]^ Pathways with a p_adj value below 0.05 were regarded as significantly enriched. Notable pathways were visualized as bar plots. Gene Ontology gene sets encompassing molecular function (MF), biological process (BP), and cellular component (CC) categories were used as reference.

### UCell Gene Set Scoring

The R package UCell v1.1.0 was used to score the gene set.^[^ ^53^ ^]^ The Mann‐Whitney U statistic was used to calculate UCell scores, which rank query genes according to how strongly they were expressed in particular cells. As UCell was a scoring method based on ranking, it was particularly well‐suited for application in large datasets that encompass multiple samples and batches.

### Cell‐Cell Communication Analysis

Cellphone DB (v2.1.0) was utilized to predict interactions between cells through known ligand‐receptor pairs. A permutation number of 1000 was chosen to calculate the null distribution for the average expression of ligand‐receptor pairs in randomized cell identities. To determine the individual expression levels of ligands or receptors, a cutoff was applied based on the average log gene expression distribution of all genes in each cell type. Significant interaction pairs, with a p‐value below 0.05 and an average log expression above 0.1, were considered important and displayed using the heatmap_plot and dot_plot functions in CellphoneDB.

### Flow Cytometric Analysis

To gate out dead cells, cells were stained according to manufacturer's instructions with Zombie NIR Fixable Viability Kit (BioLegend, 423 105). After washing, cells were stained with surface antibodies at room temperature. Cells were fixed, permeabilized, and stained for intracellular markers using True‐Nuclear Transcription Factor Fixation Buffer Set (BioLegend). Data were acquired on a BD Fortessa analyzer using FACSDiva software. Compensation and analysis were performed using FlowJo 10.7.1. The antibodies utilized for analytical flow cytometry are documented in Table S4 (Supporting Information).

### In Vitro PBMCs Culture

Freshly isolated PBMCs were stimulated using T Cell TransAct reagent (diluted at 1:100, Miltenyi Biotec, Germany) in RPMI 1640 medium (GIBCO, USA), supplemented with 10% fetal bovine serum (FBS), 1% streptomycin, and 1% penicillin (Sigma, USA), under incubation conditions of 37 °C and 5% CO^2^. Following a 48 h stimulation period, the activated PBMCs were subsequently cultured with or without the addition of 10 µg ml^−1^ pembrolizumab (PD‐1 inhibitor) (BeiGene, Beijing, China), for an additional 48 h. After this incubation period, the PBMCs were harvested and used for subsequent assays to assess T‐cell‐mediated tumor cell killing.

### T‐Cell‐Mediated Tumor Cell Killing Assay

MDA‐MB‐231 or MCF‐7 cells were plated in 24‐well plates, incubated overnight, and subsequently co‐cultured with activated T cells for 2 days at a tumor cell‐to‐T cell ratio of 1:3. Following the co‐culture, the plates were washed with PBS to eliminate cellular debris and T cells. The surviving cells were then stained with crystal violet.

### Statistical Analysis

Enrichment was evaluated using the Ro/e ratio, which represents the ratio between the observed cell number and the cell number expected by chi‐square test. Ro/e values greater than 1 indicated enrichment. Categorical variables were summarized using frequencies and proportions. The comparison of continuous variables between two groups was conducted using paired or unpaired Student's t‐test and Wilcoxon rank sum test. The comparison of categorical variables was achieved through the utilization of Chi‐square test or Fisher's exact test. All statistical analyses were conducted using R version 4.1.1 (https://www.r‐project.org/). A two‐sided *p* value of less than 0.05 was considered statistically significant.

## Conflict of Interest

The authors declare no conflict of interest.

## Author Contributions

D.D., X.L., and H.Z. contributed equally to this work. B.C. and X.L. contributed to the study's design. X.L. and Y.L. responsible for clinical sample collection. D.D., H.Z., Y.L., L.C., C.L., J.Z., Y.W., Y.L., and B.C. carried out the research and analyzed the results. D.D., Y.L., H.Z., and B.C. contributed to the method of the study. D.D., Y.L., H.Z., H.T., and B.C. supervised the study, wrote and revised the manuscript.

## Supporting information



Supporting Information

Supplemental Table 1

Supplemental Table 2

Supplemental Table 3

Supplemental Table 4

## Data Availability

The data that support the findings of this study are available from the corresponding author upon reasonable request.
