# Supplementary material for: Landscape of the Peripheral Immune Response Induced by Intraoperative Radiotherapy Combined with Surgery in Early Breast Cancer Patients
Source: Adv Sci (Weinh). 2024 Nov 4;12(1):2308174. doi: 10.1002/advs.202308174 (PMC11714210; doi:10.1002/advs.202308174)
Supplement: Supplementary file 1 — Supporting Information [file ADVS-12-2308174-s005.pdf]

ADVANCED SCIENCE
Open Access

# Supporting Information

for *Adv. Sci.*, DOI 10.1002/advs.202308174

Landscape of the Peripheral Immune Response Induced by Intraoperative Radiotherapy Combined with Surgery in Early Breast Cancer Patients

*Danian Dai, Xuerui Li, Hongkai Zhuang, Yun Ling, Lezi Chen, Cheng Long, Jinhui Zhang, Yunjie Wang, Yuehua Li*, Hailin Tang* and Bo Chen**

Figure S1

A

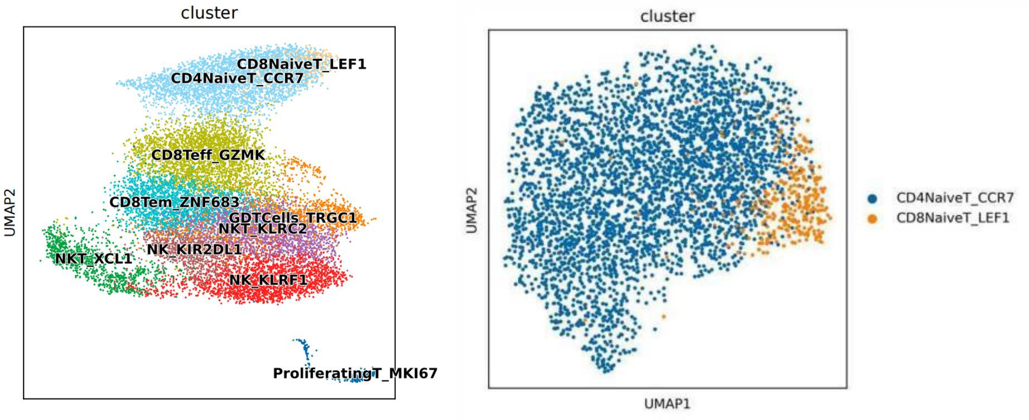


B

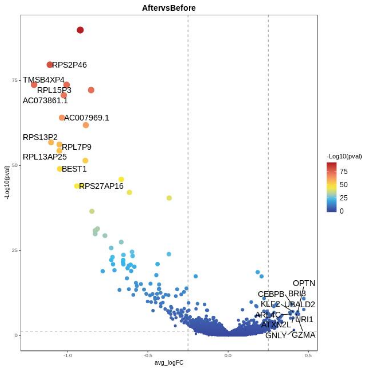


C

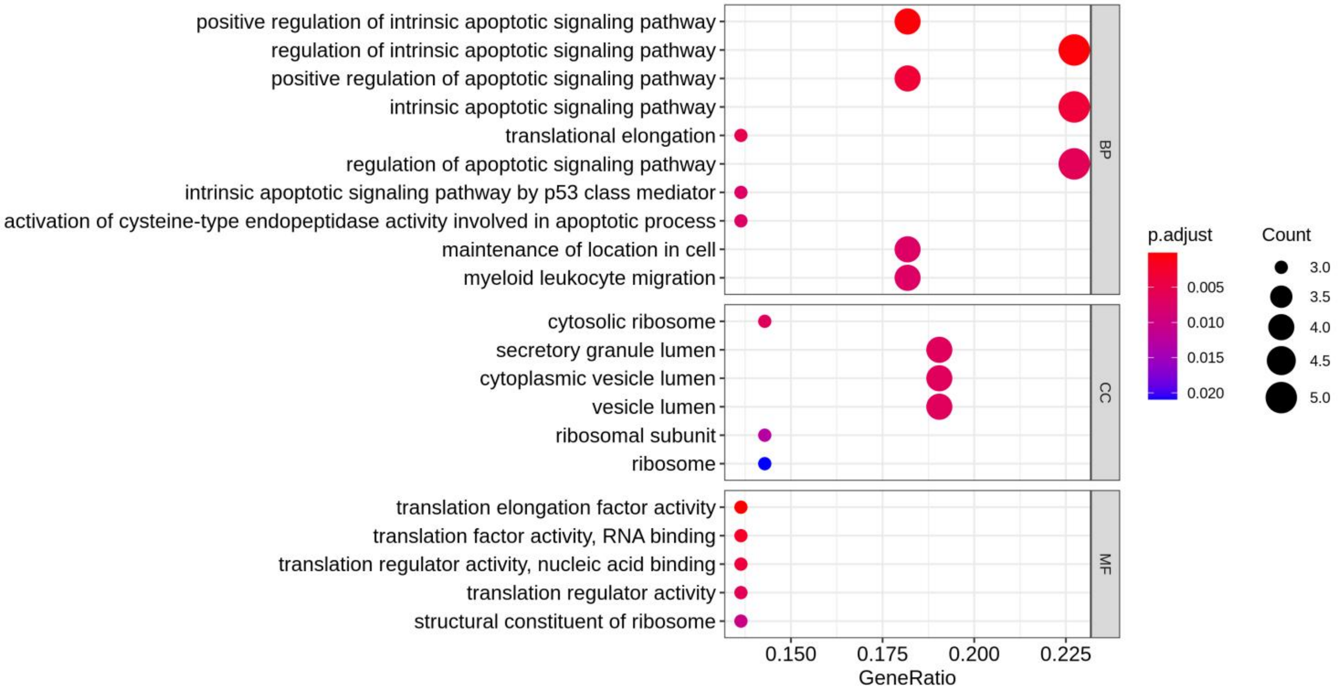

Figure S2

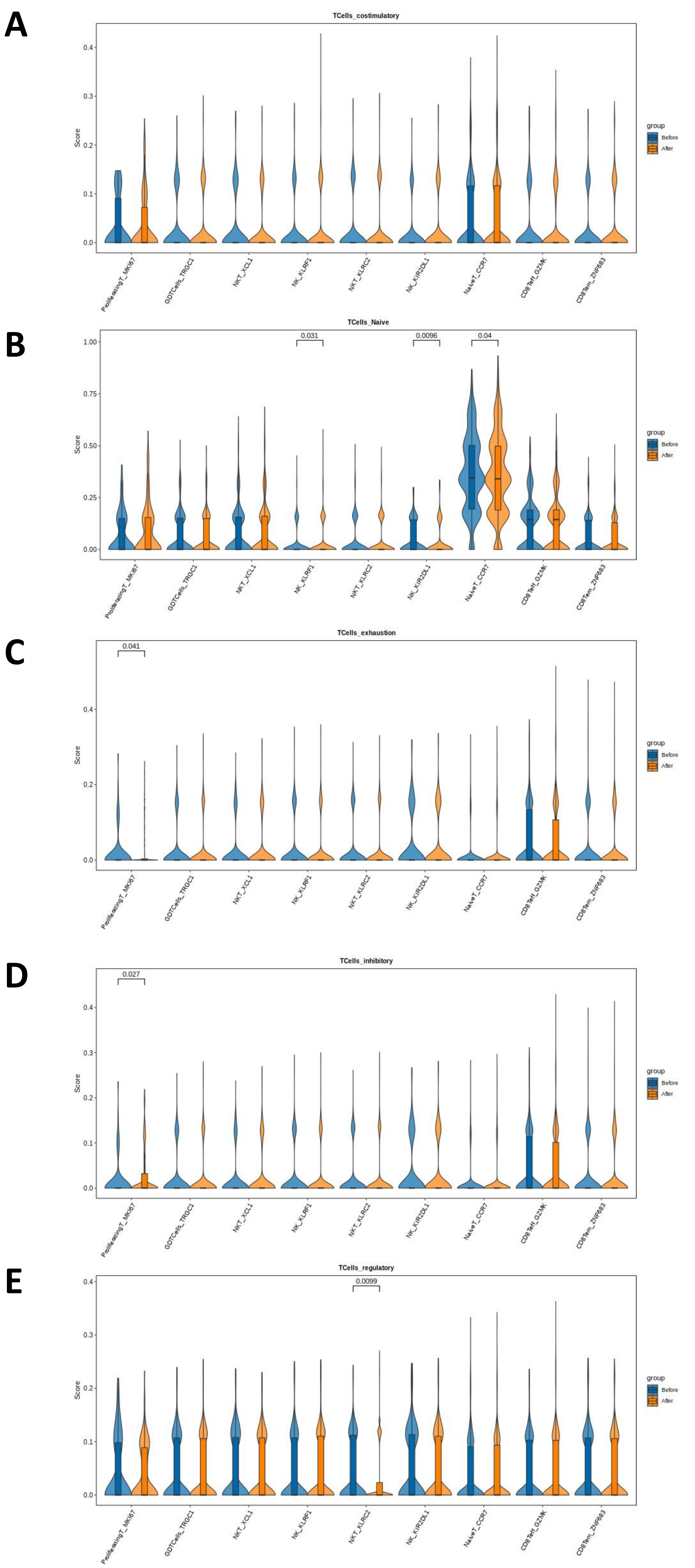
A
TCells_costimulatory
B
TCells_Naive
0.031
0.0096
0.04
C
TCells_exhaustion
0.041
D
TCells_inhibitory
0.027
E
TCells_regulatory
0.0099
Score
group
Before
After
ProliferatingT_MKI67
GDTCells_TRGC1
NKT_XCL1
NK_KLRF1
NKT_KLRC2
NK_KIR2DL1
NaiveT_CCR7
CD8Teff_GZMK
CD8Tem_ZNF683

Figure S3

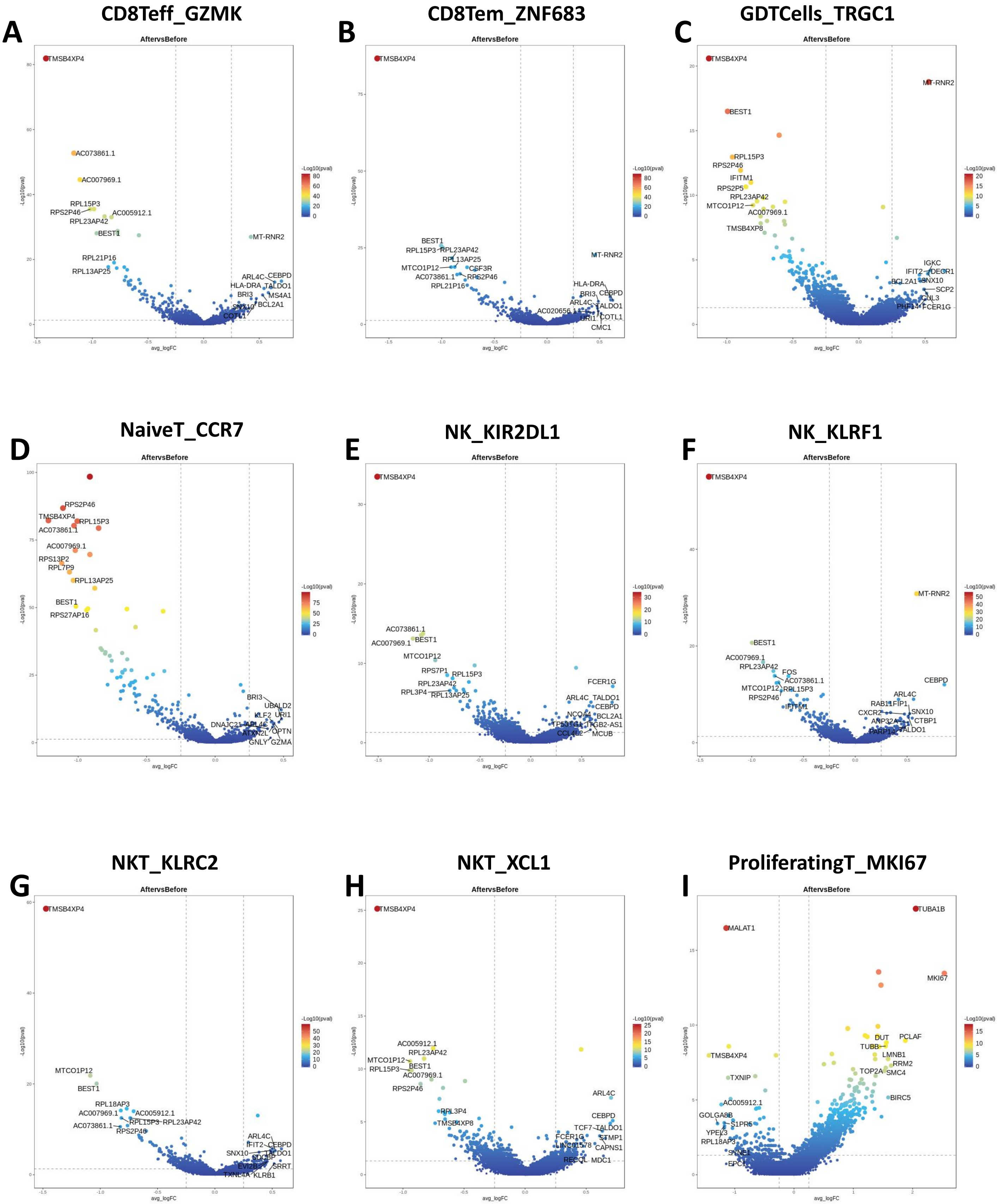
A
CD8Teff_GZMK
AftervsBefore
TMSB4XP4
AC073861.1
AC007969.1
RPL15P3
RPS2P46
AC005912.1
RPL23AP42
BEST1
MT-RNR2
RPL21P16
RPL13AP25
ARL4C
CEBPD
HLA-DRA
TALDO1
BRI3
MS4A1
BCL2A1
SNX10
COTL1
-Log10(pval)
avg_logFC
B
CD8Tem_ZNF683
AftervsBefore
TMSB4XP4
BEST1
RPL15P3
RPL23AP42
RPL13AP25
MTCO1P12
CSF3R
AC073861.1
RPS2P46
RPL21P16
MT-RNR2
HLA-DRA
BRI3
CEBPD
ARL4C
AC020656.1
TALDO1
URI1
COTL1
CMC1
C
GDTCells_TRGC1
AftervsBefore
TMSB4XP4
MT-RNR2
BEST1
RPL15P3
RPS2P46
IFITM1
RPS2P5
RPL23AP42
MTCO1P12
AC007969.1
TMSB4XP8
IGKC
IFIT2
DECR1
BCL2A1
SNX10
SCP2
CUL3
PHF14
FCER1G
D
NaiveT_CCR7
AftervsBefore
RPS2P46
TMSB4XP4
RPL15P3
AC073861.1
AC007969.1
RPS13P2
RPL7P9
RPL13AP25
BEST1
RPS27AP16
BRI3
UBALD2
KLF2
URI1
DNAJC21
ARL4C
ATXN2L
OPTN
GNLY
GZMA
E
NK_KIR2DL1
AftervsBefore
TMSB4XP4
AC073861.1
AC007969.1
BEST1
MTCO1P12
RPS7P1
RPL15P3
RPL23AP42
RPL3P4
RPL13AP25
FCER1G
ARL4C
TALDO1
CEBPD
NCOA4
BCL2A1
TP53TG1
ITGB2-AS1
CCL4L2
MCUB
F
NK_KLRF1
AftervsBefore
TMSB4XP4
MT-RNR2
BEST1
AC007969.1
RPL23AP42
FOS
AC073861.1
MTCO1P12
RPL15P3
RPS2P46
IFITM1
CEBPD
ARL4C
RAB11FIP1
CXCR2
SNX10
ANP32A
CTBP1
PARP10
TALDO1
G
NKT_KLRC2
AftervsBefore
TMSB4XP4
MTCO1P12
BEST1
RPL18AP3
AC007969.1
AC005912.1
RPL15P3
RPL23AP42
AC073861.1
RPS2P46
ARL4C
IFIT2
CEBPD
SNX10
TALDO1
SDCBP
EVI2B
SRRT
TXNL4A
KLRB1
H
NKT_XCL1
AftervsBefore
TMSB4XP4
AC005912.1
RPL23AP42
MTCO1P12
BEST1
RPL15P3
AC007969.1
RPS2P46
RPL3P4
TMSB4XP8
ARL4C
CEBPD
TCF7
TALDO1
FCER1G
STMP1
LINC01578
CAPNS1
RECQL
MDC1
I
ProliferatingT_MKI67
AftervsBefore
TUBA1B
MALAT1
MKI67
PCLAF
DUT
TUBB
TMSB4XP4
LMNB1
RRM2
TOP2A
SMC4
TXNIP
BIRC5
AC005912.1
GOLGA8B
S1PR5
YPEL3
RPL18AP3
SYNE1
EPC1

# Figure S4

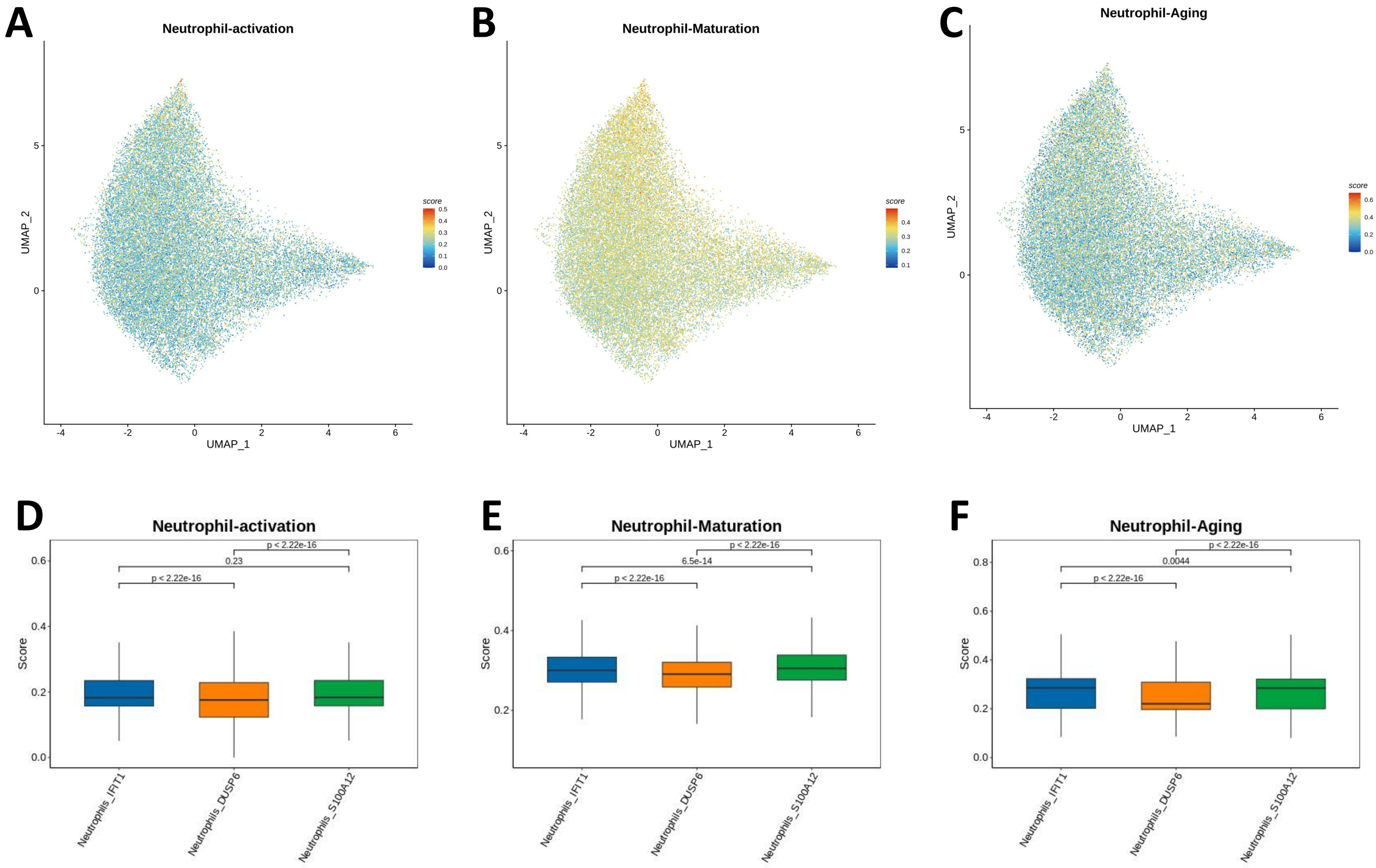

A
Neutrophil-activation
B
Neutrophil-Maturation
C
Neutrophil-Aging
UMAP_1
UMAP_2
score
D
Neutrophil-activation
E
Neutrophil-Maturation
F
Neutrophil-Aging
Score
p < 2.22e-16
0.23
6.5e-14
0.0044
Neutrophils_IFIT1
Neutrophils_DUSP6
Neutrophils_S100A12

Figure S5

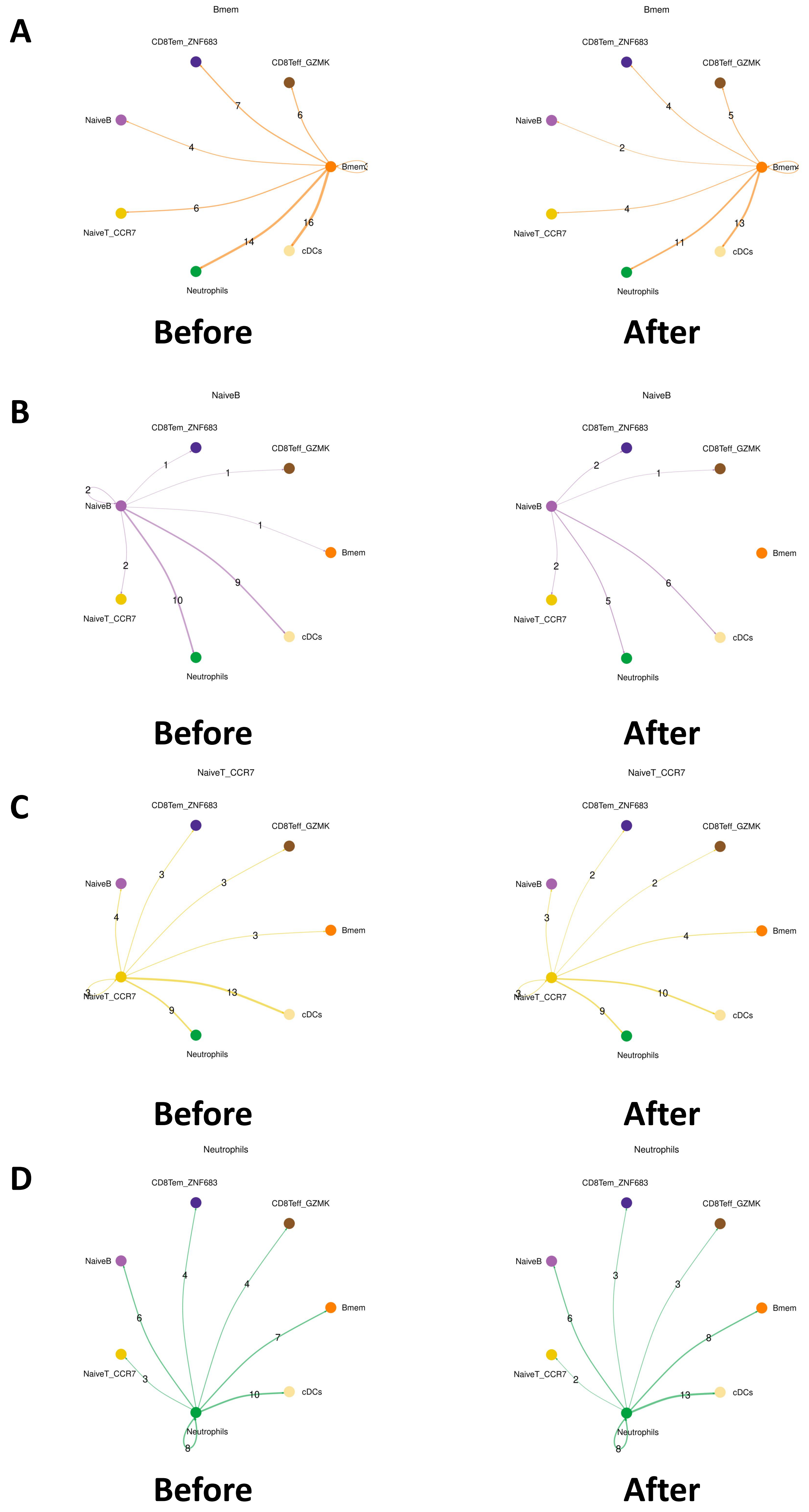
A
Bmem
CD8Tem_ZNF683
CD8Teff_GZMK
NaiveB
NaiveT_CCR7
cDCs
Neutrophils
Before
After
B
NaiveB
Before
After
C
NaiveT_CCR7
Before
After
D
Neutrophils
Before
After
